# Exploring the association between inflammatory biomarkers and gastric cancer development: A two-sample mendelian randomization analysis.

**DOI:** 10.1097/MD.0000000000036458

**Published:** 2023-02-02

**Authors:** Wenjing Yang, Ye Lv, Tao Ma, Ningju Wang, Ping Chen, Quanxia Liu, Hui Yan

**Affiliations:** a General Hospital of Ningxia Medical University, Yinchuan, China.

**Keywords:** fatty acid-binding protein 4, gastric cancer, inflammatory biomarkers, interleukin 6 receptor, Mendelian randomization

## Abstract

This study aimed to elucidate the potential causative links between inflammatory biomarkers and gastric cancer risk via a two-sample Mendelian randomization approach. Leveraging genome-wide association study (GWAS) data, we conducted a two-sample Mendelian randomization analysis. Instrumental variable selection for inflammatory markers – namely, tissue factor, monocyte chemotactic protein-1, E-selectin, interleukin 6 receptor, and fatty acid-binding protein 4 – was informed by SNP data from the IEU database. Strongly associated SNPs served as instrumental variables. We applied a suite of statistical methods, including Inverse Variance Weighted (IVW), Weighted Median Estimator (WME), MR-Egger, and mode-based estimates, to compute the odds ratios (ORs) that articulate the impact of these markers on gastric cancer susceptibility. The IVW method revealed that the interleukin 6 receptor was inversely correlated with gastric cancer progression (OR = 0.86, 95% CI = 0.74–0.99, *P* = .03), whereas fatty acid-binding protein 4 was found to elevate the risk (OR = 1.21, 95% CI = 1.05–1.39, *P* = .03). Instrumental variables comprised 5, 4, 7, 2, and 3 SNPs respectively. Convergent findings from WME, MR-Egger, and mode-based analyses corroborated these associations. Sensitivity checks, including heterogeneity, horizontal pleiotropy assessments, and leave-one-out diagnostics, affirmed the robustness and reliability of our instruments across diverse gastric malignancy tissues without substantial bias. Our research suggests that the interleukin 6 receptor potentially mitigates, while fatty acid-binding protein 4 may contribute to the pathogenesis of gastric cancer (GC). Unraveling the intricate biological interplay between inflammation and oncogenesis offers valuable insights for preemptive strategies and therapeutic interventions in gastric malignancy management.

**Highlight Box**

**Key Findings**

- The study found a causal relationship between inflammatory factors and the risk of gastric malignancy. Specifically, interleukin 6 receptor decreased the incidence of gastric malignancies, while fatty acid binding protein 4 increased the incidence.

**What is Known and What is New?**

- Inflammatory factors and chronic gastritis are key contributors to gastric malignancy.
- This study identifies interleukin 6 receptor and fatty acid binding protein 4 as specific inflammatory factors influencing the development of gastric malignancies.

**What is the Implication, and What Should Change Now?**

- These findings suggest that understanding and intervening in the inflammatory response and associated inflammatory factors may help prevent and treat gastric malignancies. Further research is needed to explore the relationship between inflammatory factors and the pathogenesis of gastric malignancies, potentially leading to new therapeutic targets and strategies.

## 1. Introduction

Stomach cancer, known for its alarming prevalence and mortality, stands as the fifth most diagnosed malignancy globally and occupies the third position in cancer-related mortality as per the World Health Organization.^[1,2]^ The geographic disparity in incidence rates is pronounced, with East Asian nations like China, Japan, and South Korea experiencing heightened rates,^[3,4]^ in stark contrast to the lower incidences observed in North America and select European regions. The male population bears a disproportionately higher burden of this disease,^[5–7]^ which may stem from gender-specific variations in diet, lifestyle, and genetic factors. Furthermore, the age-related trends in gastric cancer are noteworthy, with incidences predominantly escalating in individuals over the age of 50, affecting mainly the middle-aged and senior demographics. The complications associated with this cancer type, such as local invasion, metastasis, and gastrointestinal disturbances like bleeding, obstruction, and perforation, are of significant clinical concern.^[8–10]^ The infiltrative growth nature of stomach cancer facilitates the tumor’s encroachment into adjacent tissues, often leading to severe outcomes including bleeding and gastric perforation.^[11]^ Metastatic spread, primarily through lymphatics and the bloodstream to organs such as the liver, lungs, and bones, exacerbates organ dysfunction, severely diminishing affected patients’ life quality.^[12]^

Inflammation, a nonspecific defense mechanism, arises from tissue injury, infection, or irritation and is characterized by a multifaceted physiological cascade that includes various cell types, cytokines, and chemical messengers.^[13]^ The primary objectives of inflammation are to eliminate pathogens, repair the afflicted tissues, and establish conditions conducive to healing. Nevertheless, if inflammation is protracted or intense, it may inadvertently cause further tissue damage and propel disease advancement.^[14–16]^The association between inflammation and gastric cancer is particularly noteworthy. Persistent gastritis and infections within the gastric environment, notably those instigated by *Helicobacter pylori (H pylori*), are acknowledged as significant precursors to gastric malignancy. The chronic inflammation resulting from *H pylori* can precipitate alterations in the gastric mucosa both structurally and functionally, which may catalyze abnormal cellular proliferation and the onset of carcinogenesis. Inflammatory mediators, including interleukin 1β (IL-1β), interleukin-6 (IL-6), and tumor necrosis factor-α, which are prevalent in chronic gastritis, are implicated in promoting cellular proliferation, thwarting programmed cell death, stimulating new blood vessel formation, and fostering genetic instability – all of which can contribute to the progression of gastric tumors.^[17]^ Moreover, certain molecules and cytokines involved in the inflammatory pathway are intimately linked to the invasiveness and metastatic behavior of gastric cancers.^[18]^ For instance, transforming growth factor-β (TGF-β), which is upregulated in inflammatory states, may enhance the metastatic potential of cancer cells. Additionally, inflammatory cells upon activation release a spectrum of chemokines and proteases, further aiding the invasive and metastatic capabilities of tumor cells.^[19]^

E-selectin, monocyte chemotactic protein-1 (MCP-1), tissue factor, interleukin 6 receptor (IL-6R), and fatty acid-binding protein 4 (FABP4) are biomarkers intricately linked to the inflammatory cascade, each playing distinct yet interconnected roles in the body’s inflammatory response, particularly in the context of gastric malignancies.^[20–24]^ While their specific functions and mechanisms of action vary, there are critical areas of overlap, notably in their collective response to inflammation in gastric cancer. Initially, all these biomarkers are synthesized as part of the inflammatory response, a fundamental immune reaction to injury, infection, or other stimuli that involves an array of cells and molecules.^[25–27]^ E-selectins serve as cellular adhesion molecules on endothelial cells, facilitating the adherence of leukocytes during inflammation. MCP-1 is a key chemokine that orchestrates the migration of monocytes and macrophages to the inflammatory site. Tissue factor is implicated in the modulation of both inflammation and immune responses, serving as a cytokine.^[28–30]^ The IL-6 receptor plays a pivotal role in mediating the actions of IL-6, which is multifaceted in inflammation. FABP4, a lipid-binding protein, is linked with both inflammatory and metabolic dysregulation. Moreover, in the milieu of gastric cancer – a disease marked by pronounced heterogeneity and a strong inflammatory component – there is a noted alteration in the expression and regulatory dynamics of these markers.^[31]^ Typically, the levels of E-selectin, MCP-1, tissue factor, IL-6R, and FABP4 are elevated in gastric cancer tissues, with their aberrant expression closely tied to tumor proliferation, invasion, metastasis, and overall patient prognosis. These biomarkers can influence the trajectory of gastric malignancies in various capacities, such as enhancing the infiltration of inflammatory cells, fostering tumor angiogenesis, and modulating immune responses.^[32,33]^Furthermore, these inflammatory biomarkers are not isolated in their activity but are part of a complex network of interactions. Inflammatory stimuli, for instance, can augment the expression of E-selectin on endothelial cells, subsequently recruiting leukocytes. MCP-1 facilitates monocyte and macrophage migration, amplifying inflammatory processes. The signaling pathways involving tissue factor and IL-6R can mutually influence one another, thus intensifying the activation of inflammatory cells and cytokine release.^[34,35]^ FABP4, integral to both fatty acid metabolism and the inflammatory response, interacts with these markers, contributing to the broader inflammatory milieu.^[36,37]^

The Mendelian randomization approach is an increasingly utilized methodological design in medical research, particularly pertinent in discerning the complex interplay between inflammation and gastric carcinogenesis. This design leverages genetic variants as instrumental variables to mitigate confounding influences, thereby elucidating the causal associations inherent to this interplay.^[38–40]^ In essence, Mendelian randomization acts as a surrogate for randomized controlled trials, offering a robust framework for assessing causality in observational data. This investigative method has proven to be instrumental in enhancing our comprehension of the intricate mechanisms whereby inflammatory processes may potentiate the development of gastric cancer. Through the examination of genetic predispositions linked to inflammation, deeper insights into the causative links between inflammatory states and gastric carcinogenesis are revealed. This not only augments our understanding but also furnishes potential targets and strategic avenues for both prophylaxis and therapeutic intervention in gastric cancer.^[41]^Despite the insights garnered, it is imperative for forthcoming research endeavors to delve into the roles of additional inflammatory mediators. A comprehensive grasp of these mechanisms is vital for a holistic understanding of how inflammation influences gastric cancer pathogenesis.^[42–44]^ The objective of the present study was to utilize a two-sample Mendelian randomization analysis, incorporating data from genome-wide association studies (GWAS), to probe the causal nexus between inflammatory biomarkers and the risk of gastric malignancy. By doing so, it seeks to uncover innovative avenues for the early prevention and management of GC, while also providing empirical guidance for the clinical treatment of gastric malignancy.

## 2. Materials and methods

### 2.1. Study design

The current investigation employs a two-sample Mendelian randomization methodology to delineate the causal connections between inflammatory markers and the risk of gastric malignancy development. Commencing with the procurement of single nucleotide polymorphisms (SNPs) that correlate with both inflammatory markers (serving as instrumental variables) and gastric malignancy (as the outcome variable) from the IEU OpenGWAS database, a meticulous selection of instrumental variables (IVs) is conducted. This process utilizes a suite of statistical methods – Inverse Variance Weighted (IVW), Weighted Median Estimator, MR-Egger, Simple Mode, and Weighted Mode. These analytical techniques are integral in the appraisal of the causal inference, quantified by odds ratios (ORs), between the levels of inflammatory markers and the susceptibility to gastric malignancy.

### 2.2. Data sources

The compilation of datasets pertinent to inflammatory markers and gastric malignancy risk was sourced from the Integrative Epidemiology Unit (IEU) OpenGWAS database. This comprehensive repository amalgamates a staggering 42,347 datasets from the GWAS catalog, which includes an array of 245,542,320,999 genetic associations (accessible at http://gwas.mrcieu.ac.uk/datasets).

For the specific context of gastric malignancies, the GWAS dataset employed was derived from the research of O’Mara TA et al, encompassing a participant cohort of 218,792 individuals. Within this cohort, 633 cases were identified as patients with gastric malignancies, while a comparison group consisting of 218,159 individuals were categorized as non-gastric malignancy cases. This study reported on a substantial volume of 16,380,466 SNPs. Further granularity is provided for specific inflammatory markers, with monocyte chemotactic protein-1 levels being analyzed across a sample of 21,758 individuals, represented by 13,137,212 SNPs. Similarly, the tissue factor levels were scrutinized within the same sample size, yielding 13,098,661 SNPs. Interleukin 6 receptor and fatty acid binding protein 4 each had their expression levels and genetic associations studied within a smaller cohort of 3394 samples, sharing an identical pool of 5270,646 SNPs. These datasets underpin the subsequent analyses and contribute significantly to the robustness of the Mendelian randomization approach employed in this study. Detailed tabulation of these datasets is provided in Table 1 of the publication for an extensive review.

**Table 1:** Summary information of the genome-wide association study data on exposure and outcome in this two-sample mendelian randomization study.

Exposures/outcomes	Data source	Population	Sample size	SNP	Year
Gastric malignancies	IEU	European	218,792	16,380,466	2021
E-selectin	IEU	European	21,785	13,131,998	2020
Monocyte Chemoattractant Protein-1	IEU	European	21,758	13,137,212	2020
Tissue factor levels	IEU	European	21,758	13,098,661	2020
Interleukin 6 receptor	IEU	European	3394	5,270,646	2018
Fatty acid-binding protein 4	IEU	European	3394	5,270,646	2018

SNP = single nucleotide polymorphic.

In a Mendelian Randomization (MR) study, instrumental variables (IVs) are genetic variants used as proxies for a modifiable exposure (such as inflammatory indicators) to test for causal relationships with an outcome (like gastric malignancy). The IVs should satisfy 3 core assumptions: Association Hypothesis: The IVs must be strongly associated with the exposure. This means the chosen genetic variants should correlate significantly with the inflammatory markers being studied. Independence Hypothesis: The IVs must be independent of any confounders. The genetic variants used as IVs should not be associated with factors that could confound the exposure-outcome relationship. Exclusivity Hypothesis: The IVs affect the outcome only through the exposure, not through any other pathway.

For the selection of IVs, Single nucleotide polymorphisms (SNPs) that reach a genome-wide significance level (typically a *P* value < 5 × 10^−8^) are selected. To ensure independence among SNPs, a linkage disequilibrium (LD) clumping is performed, often with parameters such as *r*^2^ < 0.001 and a distance of 10,000 kilobases, to remove SNPs that are in LD with each other. SNPs with F-statistics greater than 10 are selected as this indicates a strong association with the exposure and reduces the risk of weak instrument bias. Table 2 in the study would typically present the selected SNPs for each inflammatory marker, detailing the chromosome location, the associated gene, effect allele, minor allele frequency (MAF), beta coefficients, and *P* values for the association with the exposure (inflammatory markers use in the given context), as well as the F-statistics for each SNP locus. This process aims to ensure that the instrumental variables used in the MR analysis are valid and reliable for testing the hypothesized causal effect of inflammatory markers on the risk of gastric malignancy.

**Table 2:** Mendelian randomization of inflammatory factors on gastric malignancies.

Exposure	MR method	SNP	F	Or (or_95% CI)	SE	*P* value
ebi-a-GCST90012000 (E-selectin)	MR Egger	7	76.23	0.722 (0.480–1.084)	0.21	.18
	Inverse variance weighted	7		0.810 (0.608–1.079)	0.16	.15
	Weighted mode	7		0.762 (0.537–1.081)	0.15	.19
ebi-a-GCST90012007 (Monocyte Chemoattractant Protein-1)	MR Egger	7	45.67	6.406 (0.041–1009.025)	0.21	.55
	Inverse variance weighted	7		1.238 (0.511–2.999)	0.16	.64
	Weighted mode	7		2.416 (0.421–13.851)	0.15	.40
ebi-a-GCST90012014 (Tissue factor levels)	MR Egger	5	73.49	0.745 (0.264–2.101)	0.53	.62
	Inverse variance weighted	5		0.747 (0.514–1.086)	0.22	.13
	Weighted mode	5		0.746 (0.467–1.191)	0.19	.29
prot-b-23 (Interleukin 6 receptor)	Inverse variance weighted	2	42.15	0.857 (0.742–0.989)	0.07	.04
prot-b-71 (Fatty acid-binding protein 4)	MR Egger	3	33.41	0.897 (0.476–1.690)	0.32	.79
	Inverse variance weighted	3		1.214 (1.002–1.470)	0.11	.04
	Weighted mode	3		1.178 (0.921–1.506)	0.10	.32

MR = mendelian randomization.

### 2.3. Methodology of causality validation

In this study, the primary method for assessing causality between inflammatory markers and GC risk was the IVW model. Supplementary methods, such as Weighted Median Estimator, MR Egger, and others, were employed for result validation. Heterogeneity was checked using Cochran’s Q test, and horizontal pleiotropy was assessed with MR-PRESSO and MR-Egger tests. Sensitivity was analyzed via the Leave-one-out approach. All analyses were conducted using the Two Sample MR and MRPRESSO packages in R version 4.2.3.

## 3. Results

To examine the causal connections between inflammatory markers and gastric malignancy, our analysis utilized 5, 4, 7, 2, and 3 single nucleotide polymorphisms (SNPs) as instrumental variables, respectively. These SNPs were related to levels of tissue factor, monocyte chemotactic protein-1, E selectin, interleukin 6 receptor, and fatty acid binding protein 4.

The influence of inflammatory mediators on gastric cancer risk is presented in Table 2. Notably, the interleukin 6 receptor was associated with a reduced incidence of gastric malignancies (Odds Ratio [OR] = 0.86, 95% Confidence Interval [CI] = 0.74–0.99, *P* value = .03, using the IVW method). In contrast, fatty acid binding protein 4 was found to increase the risk of gastric malignancies (OR = 1.01, 95% CI = 1.00–1.02, *P* value = .03, using the IVW method). These findings were consistent across the Weighted Median Estimator, Weighted mode, and Simple mode methodologies. However, MR Egger’s analysis did not show a statistically significant association between fatty acid binding protein 4 levels and the risk of gastric malignancy (OR = 0.897, 95% CI = 0.476–1.690, *P* value = .79).

**Figure 1.**
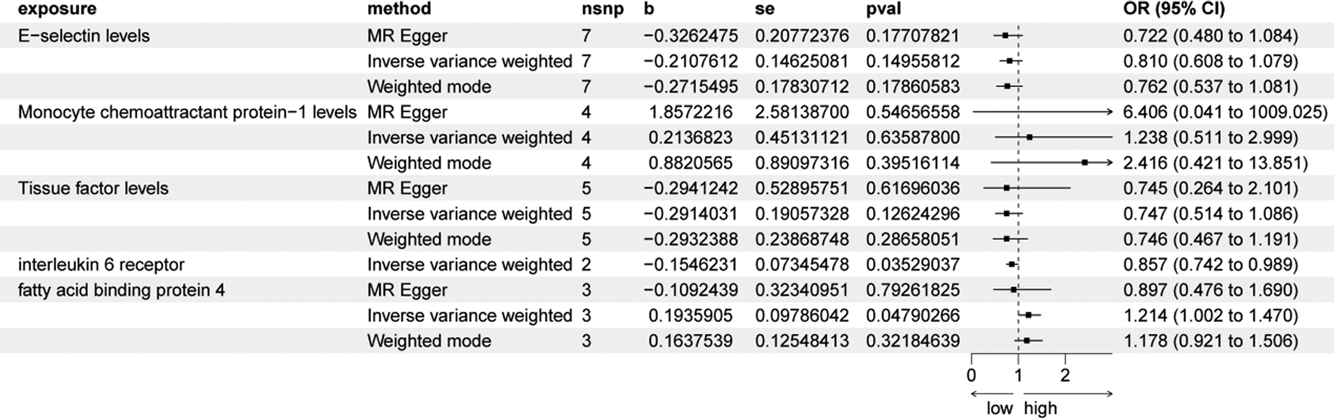
Forest plot of inflammatory factors on the risk of developing gastric malignancies. Dots and bars represent causal estimates of the risk of inflammatory factors with developing gastric malignancies. CI = confidence interval, OR = odds ratio, SNP = single-nucleotide polymorphism.

The forest plot illustrated a noteworthy causal relationship between both the interleukin 6 receptor and fatty acid binding protein 4 with gastric malignancy risk, suggesting the interleukin 6 receptor may have a protective effect, whereas fatty acid binding protein 4 appears to increase susceptibility to gastric malignancy (Fig. 1).

**Figure 2.**
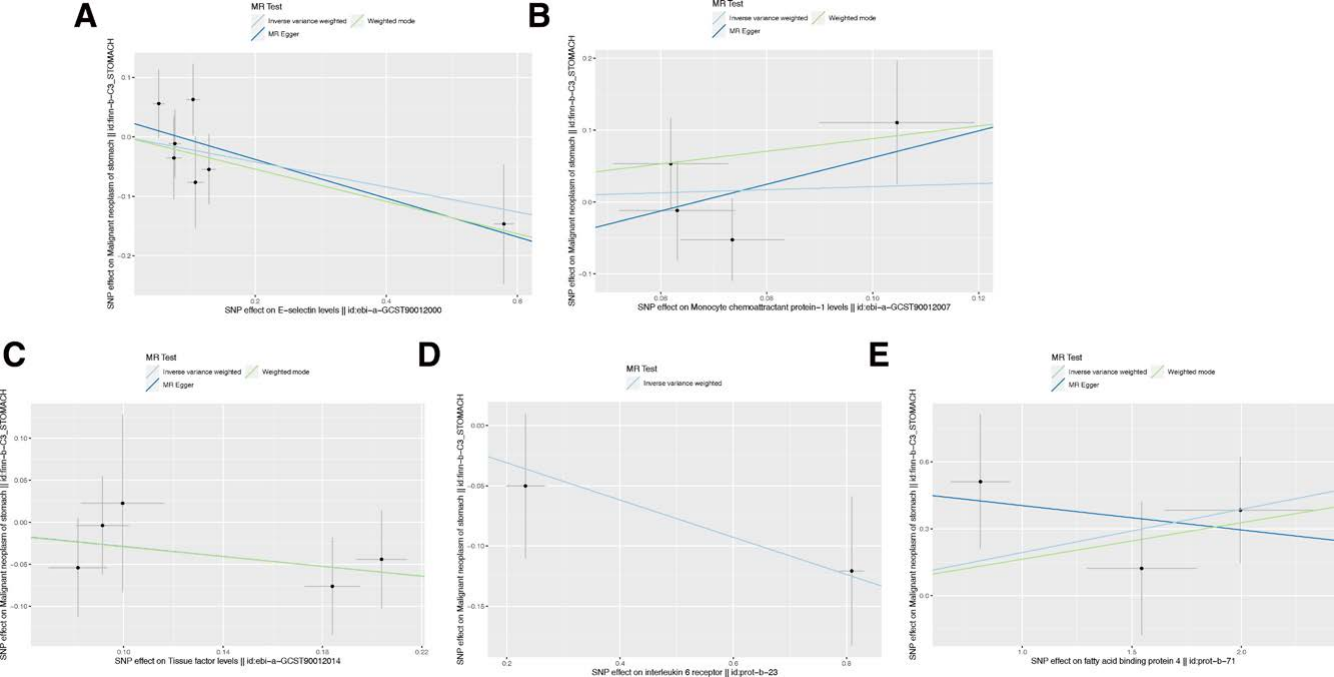
Scatter plot of the association between inflammatory indicators and the risk of gastric malignancy. (A) E-selectin; (B) MCP-1; (C) tissue factor; (D) IL-6 receptor; (E) FABP4. Each black dot represents a SNP, plotted from the SNP estimate for inflammatory indicators and the SNP estimate for gastric malignancy risk, with a standard error bar. The slope of the line corresponds to a causal estimate using each of the different methods. SNP = single nucleotide polymorphism.

**Figure 3.**
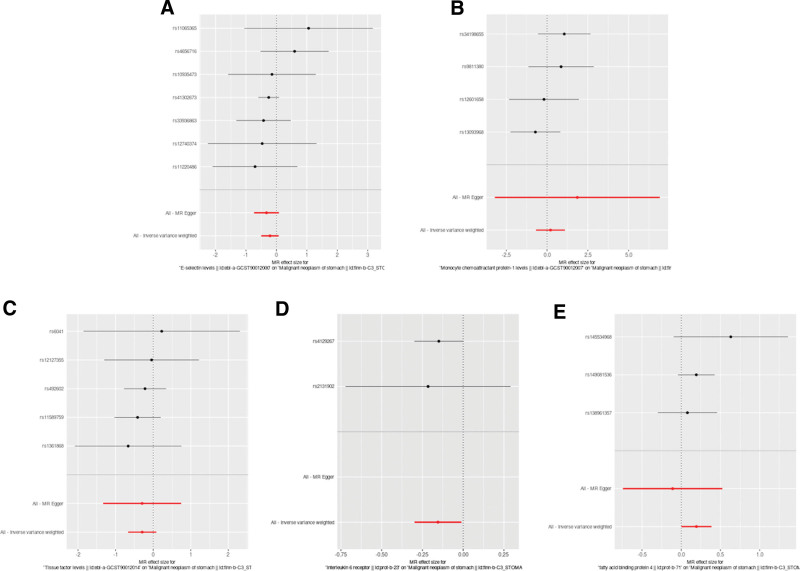
Forest plot of the association between inflammatory indicators and the risk of gastric malignancy. (A) E-selectin; (B) MCP-1; (C) tissue factor; (D) interleukin 6 receptor; (E) FABP4. Dots and bars represent causal estimates of the risk of gastric malignancy with inflammatory indicators.

**Figure 4.**
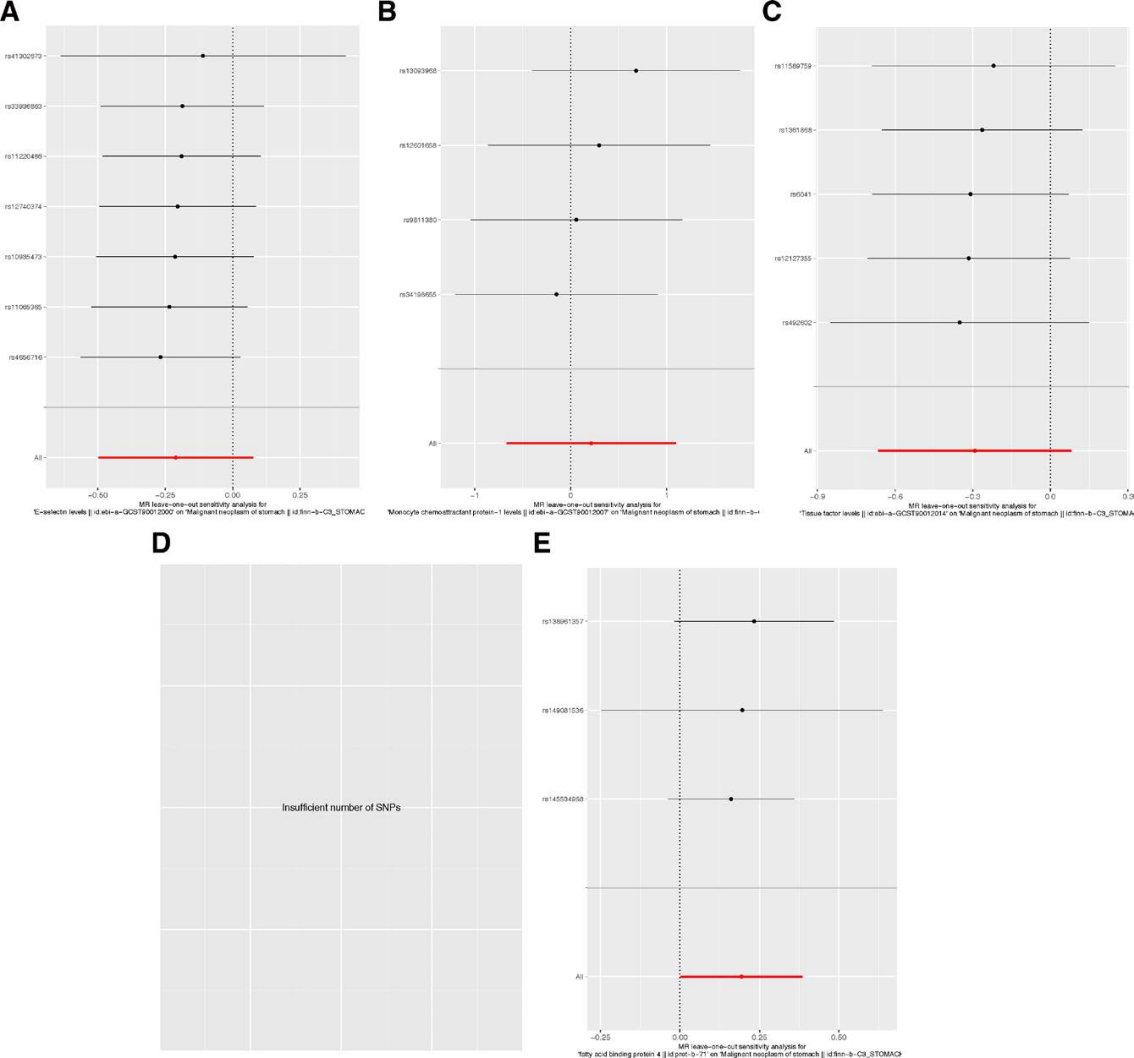
Leave-one-out sensitivity analysis of the association between inflammatory indicators and gastric malignancy. (A) E-selectin; (B) MCP-1; (C) tissue factor; (D) FABP4. The dot and bar indicate the estimates and 95% confidence interval when the specific single nucleotide polymorphism is removed.

**Figure 5.**
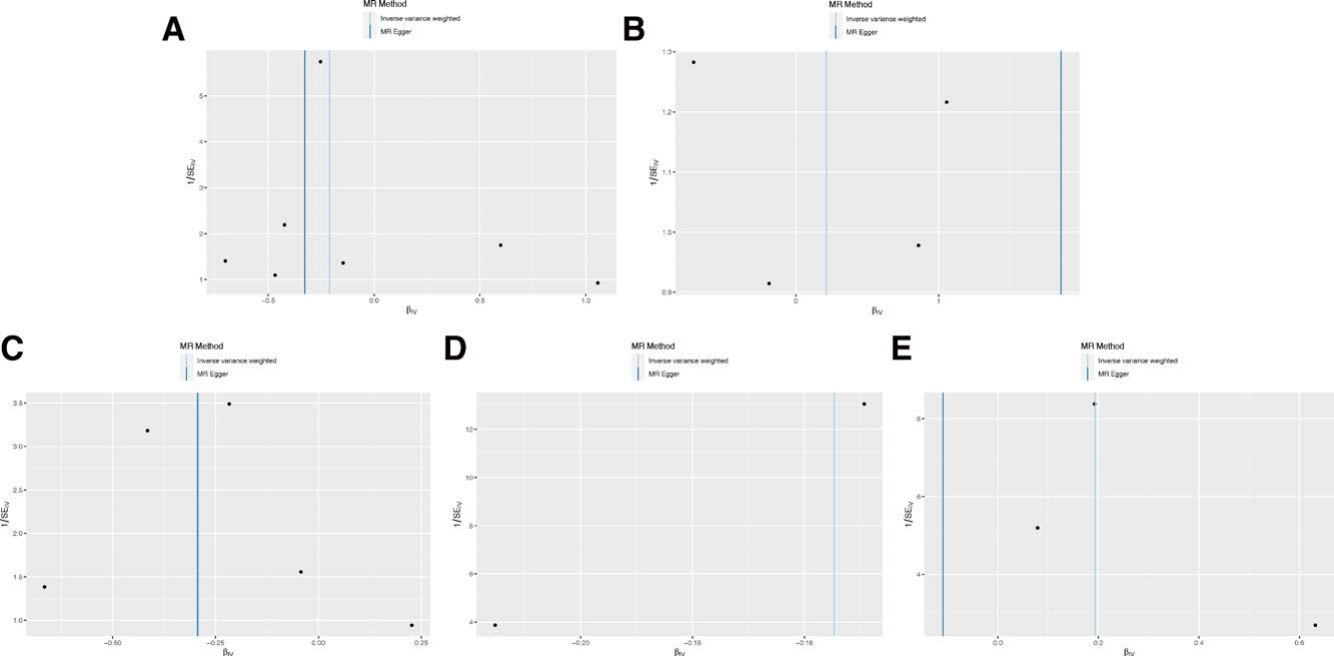
Funnel plot of the association between inflammatory indicators and the risk of gastric malignancy. (A) E-selectin; (B) MCP-1; (C) tissue factor; (D) IL-6 receptor; (E) FABP4. Each black dot indicates a single nucleotide polymorphism.

Upon conducting Cochran’s Q test, the instrumental variables (IVs) exhibited no substantial heterogeneity (*P* > .05), indicating consistency among the selected SNPs (Table 3). Further, the MR-Egger regression intercept test suggested no evidence of directional pleiotropy, as indicated by a non-significant intercept (*P* > .05), reinforcing the validity of the IVs. The study also applied the Leave-one-out sensitivity analysis to assess the influence of individual SNPs on the aggregate Mendelian Randomization (MR) estimates. The stability of the results was affirmed, with no single SNP unduly swaying the MR outcomes, as depicted in Figure 2 and the supplements to Figures 3–5. This robustness check ensures that the observed associations are not artifacts of any single genetic variant but are likely reflective of the true relationship between the inflammatory markers and gastric malignancy risk.

**Table 3:** Test of pleiotropy and heterogeneity of inflammatory factors on gastric malignancies.

	Pleiotropy test			Heterogeneity test					
	MR-Egger			MR-Egger			Inverse variance weighted		
	Intercept	SE	*P*	Q	Q_df	Q_pvalval	Q	Q_df	Q_pvalval
E-selectin	−0.124	0.191	.583	3.615	5	0.606	4.228	6	0.646
Monocyte Chemoattractant Protein-1	0.001	0.078	.996	2.482	2	0.289	3.005	3	0.391
Tissue factor levels	NA	NA	NA	0.879	3	0.830	0.879	4	0.928
Interleukin 6 receptor	0.513	0.522	.506	NA	NA	NA	0.060	1	0.806
Fatty acid-binding protein 4	0.027	0.035	.469	0.779	1	0.377	1.744	2	0.418

MR = mendelian randomization.

## 4. Discussion

In this investigation, we delved into the putative causative links between inflammatory mediators and the risk of gastric malignancy, leveraging an aggregation of extensive GWAS data alongside a two-sample Mendelian randomization framework.^[45,46]^ Our findings reveal a dichotomy in the role of specific inflammatory mediators: the interleukin 6 receptor appears to mitigate the incidence of gastric malignancies, whereas the fatty acid binding protein 4 ostensibly augments this risk.^[47,48]^ These results lend credence to the prognostic significance of inflammatory mediators concerning gastric malignancy within the context of GWAS data.

Delving into the etiology of gastric malignancy, it is evident that inflammatory factors are significant contributors. Inflammation is inherently a multifaceted biological reaction, typically serving as a defense mechanism against infections, tissue injuries, or other pathological insults. However, in instances where the inflammatory response is chronic or inordinately intense, it evolves into a pivotal etiological component of the disease progression.^[49–51]^

The correlation between inflammation and gastric malignancy has been extensively corroborated by research. On the one hand, the progression of gastric malignancy is intimately associated with chronic gastritis, a persistent inflammation of the gastric mucosa typically due to *H pylori* infection, long-term NSAID use, or other etiological factors.^[52–54]^ Chronic gastritis can precipitate a series of changes in the gastric mucosa that may culminate in intestinal metaplasia, atypical hyperplasia, and eventually, gastric malignancy. On the other hand, an imbalance in the production and regulation of inflammatory mediators is thought to play a pivotal role in the pathogenesis of gastric malignancies. Inflammatory mediators are a group of cytokines that are generated during the inflammatory response, such as E-selectin, monocyte chemotactic protein-1, tissue factor, interleukin 6 receptor, and fatty acid binding protein 4.^[55–57]^ The heightened expression of these inflammatory mediators has been closely associated with various facets of tumor initiation, growth, angiogenesis, metastasis, and resistance to chemotherapy. Inflammatory mediators contribute to the development of gastric malignancy through several mechanisms.^[58]^ Firstly, they can directly influence tumor cells, promoting their proliferation and survival. Additionally, inflammatory mediators can alter the tumor microenvironment, fostering angiogenesis and the infiltration of inflammatory cells, which provides the necessary nutrients and oxygen for tumor growth and facilitates the evasion of immune detection. Furthermore, inflammatory mediators can disrupt normal apoptosis and DNA repair pathways, leading to a decrease in the genetic stability of tumor cells. Therefore, inflammatory mediators play a substantial regulatory role in the etiology of gastric malignancies. A deeper understanding of the inflammatory response and its associated mediators may provide new avenues for the prevention and treatment of gastric malignancy.^[59–61]^ However, further research is required to fully elucidate the relationship between inflammatory mediators and the pathogenesis of gastric malignancies and to discover new therapeutic targets and approaches.^[62,63]^

Gastric malignancy is a common malignant tumor of the digestive system, and its pathogenesis involves multiple epidemiological and pathogenetic mechanisms. It occurs mostly in middle-aged and elderly people, especially in those over 50 years of age. The prevalence is higher in men than in women.^[64]^ *H pylori* is a common bacterium that infects the gastric mucosa, and long-term infection may lead to chronic gastritis and gastric ulcers, increasing the risk of gastric malignancies. In addition dietary habits, tobacco and alcohol, genetic factors and other associated factors that can cause long-term chronic inflammation have been associated with the development of gastric malignancies.^[65,66]^

Observational research has consistently documented a robust correlation between inflammatory processes and the onset of gastric malignancy.^[67]^ Various environmental and genetic evaluations underscore that heightened levels of pro-inflammatory markers correlate with an increased risk of developing gastric cancer.^[68–70]^ This concordance is further corroborated by a national, population-based matched cohort study, which posits a similar positive relationship between inflammatory biomarkers and the advent of gastric malignancy. In this context, our research presented findings that the interleukin 6 receptor curtails the risk of gastric malignancy (OR = 0.86, 95% CI = 0.74–0.99, *P* = .03, using the IVW method), a revelation that seemingly contradicts existing literature, potentially attributable to a limited sample size that could introduce bias. On the other hand, our data indicates that fatty acid binding protein 4 serves to elevate gastric malignancy risk (OR = 1.01, 95% CI = 0.74–0.99, *P* = .03, IVW method), aligning with current studies and substantiating hypotheses regarding the causal linkage between inflammatory markers and gastric malignancy pathogenesis.^[71,72]^

The study presented herein is not without its limitations. Primarily, the investigation relied on data amalgamated from the IEU database, which permitted the exploration of only the linear associations between inflammatory biomarkers and gastric malignancy, precluding the examination of any potential nonlinear dynamics. Furthermore, the dataset was confined to individuals of European descent, which may not be representative of the relationship between these variables in other ethnic cohorts. Additionally, the quantitative analysis did not extend to the specific concentrations of inflammatory markers. The broader physiological ramifications that accompany elevated inflammatory factor levels, including the resultant phenotypic alterations, warrant more exhaustive research. Moreover, while this Mendelian randomization study offers an initial appraisal of the causative link between inflammation and gastric cancer risk, the intricate underlying biological mechanisms remain to be elucidated.

In summation, the data suggests that inflammatory biomarkers may elevate the risk of developing gastric malignancies. Therein lies the potential for significant clinical impact, particularly in the realms of early prevention and therapeutic intervention, if the molecular interplay between inflammatory processes and gastric carcinogenesis can be deciphered. Such insights could enhance the predictive accuracy regarding the onset of this disease.

## 5. Conclusion

In summary, our study employed a two-sample Mendelian randomization approach to deliver genetic support for the role of interleukin 6 receptor in diminishing the risk of gastric malignancies, while fatty acid binding protein 4 appears to elevate this risk. Despite these findings, further research is imperative to corroborate the linkage between inflammatory mediators and gastric cancer risk. Subsequent investigations, including extensive randomized controlled trials, are essential to affirm the causal inferences drawn from our Mendelian randomization analysis.

## Author contributions

**Conceptualization:** Wenjing Yang, Hui Yan.

**Data curation:** Tao Ma.

**Investigation:** Ye Lv, Ningju Wang.

**Methodology:** Ping Chen, Quanxia Liu.

**Software:** Ye Lv, Ningju Wang.

**Visualization:** Ping Chen, Quanxia Liu.

**Writing – original draft:** Wenjing Yang, Ye Lv.

**Writing – review & editing:** Tao Ma, Ningju Wang, Ping Chen, Quanxia Liu, Hui Yan.
